# Paraprobiotics Derived From *Bifidobacterium animalis* Subsp. *Lactis* BB‐12 Attenuates Indomethacin‐Induced Gastric Ulcer by Inhibiting the PANoptosis Pathway

**DOI:** 10.1002/fsn3.72397

**Published:** 2026-10-07

**Authors:** Adem Yavaş, Ezgi Özkiran, Ecem Akan

**Affiliations:** ^1^ Food Quality Control and Analysis Programme, Food Processing Department, Çine Vocational School Aydin Adnan Menderes University Aydın Türkiye; ^2^ Department of Pathology Aydin Adnan Menderes University Faculty of Medicine Aydın Türkiye; ^3^ Department of Dairy Technology, Faculty of Agriculture Aydin Adnan Menderes University Aydın Türkiye

**Keywords:** gastric ulcer, inflammation, oxidant/antioxidant parameters, paraprobiotics, PANoptosis

## Abstract

Gastric ulcer pathogenesis involves inflammation and cell death. Paraprobiotics are emerging as safer therapeutic alternatives. This study aimed to investigate the gastroprotective effect of paraprobiotics derived from *Bifidobacterium animalis* subsp. *lactis* BB‐12 and its role in modulating PANoptosis in the experimental ulcer model. Gastric ulcers were induced in Balb/c mice using indomethacin. Mice were pre‐treated by oral gavage with live *B. animalis subsp. lactis* BB‐12 probiotic (1 × 10^9^ CFU/mL, 200 μL), its heat‐inactivated paraprobiotic (1 × 10^9^ CFU/mL, 200 μL), or lansoprazole (30 mg/kg), each administered 5 min before ulcer induction with indomethacin (25 mg/kg). Gastric damage was assessed macroscopically and histologically. Oxidative stress markers (superoxide dismutase [SOD], catalase [CAT], reduced glutathione [GSH], malondialdehyde [MDA]), myeloperoxidase (MPO) activity, and serum cytokines (tumor necrosis factor‐α [TNF‐α], interleukin‐6 [IL‐6], IL‐10) were measured. Key proteins of the PANoptosis pathway were analyzed by Western blot. Both probiotic and paraprobiotic treatments significantly reduced the ulcer area (from 52.54% in the ulcer group to 21.57% and 17.34%, respectively) and the total histological damage index (from 3.95 to 1.27 and 0.95, respectively) (*p* < 0.001). Both treatments reversed the depletion of antioxidant enzymes and the increase in MDA and MPO levels. Additionally, they restored IL‐10 to control levels while suppressing TNF‐α and IL‐6 (*p* < 0.001). Paraprobiotics also strongly inhibited key regulatory proteins in three cell death pathways, including NLRP3, c‐Caspase‐1/3/8, ZBP1, and p‐RIPK3/MLKL. Paraprobiotics derived from *B. lactis subsp. lactis* BB‐12 exhibit a potent gastric protective effect against gastric ulcers. As they were administered before ulcer induction, paraprobiotics represent a promising prophylactic strategy for the prevention of inflammatory gastrointestinal disorders, identifying PANoptosis as an important therapeutic target.

## Graphical abstract

Heat‐inactivated *Bifidobacterium animalis* subsp. *lactis* BB‐12 (a paraprobiotic) protects the gastric mucosa against indomethacin‐induced injury by restraining PANoptosis. In a Balb/c mouse model of indomethacin‐induced gastric ulcer, oral pre‐treatment with the live probiotic or its heat‐inactivated paraprobiotic derivative preserved mucosal integrity as effectively as—and, for several endpoints, more effectively than—the reference drug lansoprazole. Both preparations reduced the macroscopic ulcer area and histological damage, reinforced the endogenous antioxidant defense (SOD, CAT, and GSH), lowered lipid peroxidation (MDA) and neutrophil‐derived myeloperoxidase, and rebalanced the systemic cytokine profile by suppressing TNF‐α and IL‐6 while restoring IL‐10. At the molecular level, the paraprobiotic produced the most pronounced effect, coordinately inhibiting the three intersecting arms of PANoptosis: the NLRP3/caspase‐1/GSDMD–IL‐1β pyroptotic axis, the ZBP1/caspase‐8/caspase‐3/GSDME apoptotic axis, and the RIPK3/MLKL necroptotic axis. Overall, the figure illustrates how paraprobiotic BB‐12 achieves gastroprotection by simultaneously attenuating oxidative stress, inflammation, and PANoptotic cell death, supporting its potential as a safe, stable prophylactic agent and identifying PANoptosis as a promising therapeutic target.

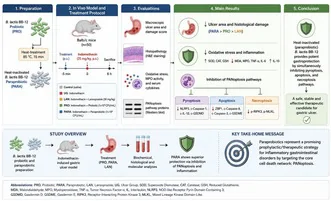

**Abbreviations**
CAT: catalase
CFU: colony‐forming unit
ELISA: enzyme‐linked immunosorbent assay
GSDMD: gasdermin D
GSDME: gasdermin E
GSH: reduced glutathione
H&E: hematoxylin and eosin
IL: interleukin
LAN: lansoprazole group
MDA: malondialdehyde
MLKL: mixed‐lineage kinase domain‐like protein
MPO: myeloperoxidase
NF‐κB: nuclear factor kappa‐B
NLRP3: NLR family pyrin domain‐containing 3
NSAID: non‐steroidal anti‐inflammatory drug
PANoptosis: integrated pyroptosis–apoptosis–necroptosis
PARA: paraprobiotic group
PI: propidium iodide
PPI: proton pump inhibitor
PRO: probiotic group
RIPK3: receptor‐interacting protein kinase 3
ROS: reactive oxygen species
SEM: standard error of the mean
SOD: superoxide dismutase
TNF‐α: tumor necrosis factor‐α
UG: ulcer group
ZBP1: Z‐DNA‐binding protein 1

## 1 Introduction

Gastric ulcer, a subtype of peptic ulcer disease, is a chronic and debilitating condition characterized by localized defects or excavations in the gastric mucosa that extend through the muscularis mucosae (Khan et al. 2023). Affecting an estimated 4 million people globally each year and with a lifetime prevalence of approximately 5%–10% in the general population, this disease represents a significant global health burden. The primary etiological factors responsible for disrupting the delicate balance between aggressive factors (e.g., gastric acid, pepsin, external agents) and cytoprotective mechanisms (e.g., the mucus‐bicarbonate barrier, prostaglandin synthesis, adequate mucosal blood flow) are chronic infection with *Helicobacter pylori* and the widespread use of non‐steroidal anti‐inflammatory drugs (NSAIDs) (Abbasi‐Kangevari et al. 2022).

Current therapeutic strategies are predominantly focused on either eradicating *H. pylori* with antibiotic regimens or reducing gastric acid secretion, the latter of which is primarily achieved using proton pump inhibitors (PPIs) and H2‐receptor antagonists (Gupta et al. 2023). While these treatments are effective in promoting ulcer healing and providing symptomatic relief, their long‐term use is associated with a growing list of significant drawbacks. For instance, prolonged PPI use has been linked to an increased risk of bone fractures, hypomagnesemia, renal complications, and gastrointestinal infections such as *Clostridium difficile* . Furthermore, the escalating problem of antibiotic resistance poses a major challenge to the efficacy of *H. pylori* eradication therapies (Gupta et al. 2023; Scarpignato and Tolone 2020). Crucially, these conventional treatments primarily address symptoms by mitigating aggressive factors but fail to target the underlying inflammatory cascade and cellular damage that perpetuate tissue injury and delay mucosal healing (Joshi et al. 2024). This therapeutic gap underscores the urgent need for novel, safer, and more comprehensive treatment strategies capable of modulating the complex pathological processes of the disease.

The pathogenesis of gastric ulceration is fundamentally an inflammation‐driven process. The initial insult to the gastric mucosa, such as that caused by NSAIDs via the inhibition of cytoprotective prostaglandins, triggers an acute inflammatory response characterized by the infiltration of immune cells, particularly neutrophils, into the lamina propria. Activated neutrophils release a battery of destructive molecules, including reactive oxygen species (ROS) and proteolytic enzymes like myeloperoxidase (MPO), leading to a state of severe oxidative stress. The excessive production of ROS overwhelms the endogenous antioxidant defense systems of the gastric mucosa, such as superoxide dismutase (SOD), catalase (CAT), and glutathione (GSH) (Ermis et al. 2023; Küçükler et al. 2022). This imbalance causes widespread lipid peroxidation, protein oxidation, and DNA damage, leading to direct injury to epithelial cells and compromising the mucosal barrier. This oxidative damage, in turn, exacerbates the inflammatory response by activating transcription factors like NF‐κB, which orchestrates the production of pro‐inflammatory cytokines such as Tumor Necrosis Factor‐alpha (TNF‐α), Interleukin‐1β (IL‐1β), and Interleukin‐6 (IL‐6) (Sayed et al. 2024; Fagundes et al. 2021). This self‐perpetuating cycle of inflammation and oxidative stress culminates in the extensive loss of gastric mucosal cells, resulting in the formation of erosions and deep ulceration. Consistent with this, recent experimental studies have confirmed that the interplay between excessive oxidative stress and pro‐inflammatory cytokine signaling is a decisive driver of mucosal injury and a key determinant of ulcer severity across chemically induced gastric ulcer models (Ahmed et al. 2026; Ahmed, Alqaisi, Ibrahim, et al. 2025; Ahmed, Alqaisi, Jabbar, et al. 2025; Al‐Qaisi et al. 2025a, 2025b).

Cell death is a critical endpoint of this pathological cascade. For years, apoptosis and necrosis were considered the primary cell death pathways in gastric ulcers (Khan et al. 2023; Jin et al. 2022). However, recent discoveries in cell biology have unveiled a more complex and regulated form of inflammatory cell death known as PANoptosis (Zhu et al. 2023). First defined in 2019, PANoptosis is a highly lytic and unique cell death pathway that integrates core molecular components from three distinct programmed cell death programs: pyroptosis, apoptosis, and necroptosis (Malireddi et al. 2019; Christgen et al. 2020). This intricate network is typically activated by pathogen‐associated or damage‐associated molecular patterns and is orchestrated by a multi‐protein cytoplasmic complex termed the “PANoptosome.” According to current mechanistic models, the PANoptosome can simultaneously activate Caspase‐1 (a hallmark of pyroptosis, leading to the cleavage of Gasdermin D and maturation of IL‐1β), Caspase‐8 (the main initiator of apoptosis), and the RIPK3‐MLKL axis (the core machinery of necroptosis) (Samir et al. 2020). Given that gastric ulcers are characterized by intense inflammation, cellular stress, and tissue damage, it likely provides an ideal microenvironment for the activation of PANoptosis. Therefore, the hypothesis that PANoptosis acts as a central driver of the massive epithelial cell loss and persistent inflammation observed in gastric ulcer pathology remains a largely unexplored area.

Targeting inflammation‐driven cell death pathways presents a promising therapeutic approach. Accordingly, there is a growing interest in identifying novel agents that can modulate the host's inflammatory response. In this context, probiotics, live microorganisms that, when administered in adequate amounts, confer a health benefit on the host, have emerged as a compelling area of research (Zommiti et al. 2020). Numerous studies have demonstrated the potential of various probiotic strains to protect the gastric mucosa through multiple mechanisms, including strengthening the epithelial barrier, competitively excluding pathogens, and most importantly, exerting potent immunomodulatory effects. Probiotics can shift the cytokine balance from a pro‐inflammatory to an anti‐inflammatory state, thereby mitigating tissue damage and promoting healing (Raheem et al. 2021; Cristofori et al. 2021; Mercado‐Monroy et al. 2025).

Despite their promise, the therapeutic application of live probiotics is not without its challenges. The primary concern is safety, especially in vulnerable populations such as immunocompromised, critically ill, or infant patients, where the risk of systemic infection or sepsis, although rare, cannot be disregarded (Borase et al. 2022). Furthermore, live probiotics face issues related to manufacturing, stability, shelf‐life, and the precise standardization of viable cell counts, which can impact their clinical efficacy and reproducibility (Wendel 2022). A paradigm shift has occurred with the growing understanding that the beneficial effects of probiotics are not always contingent on their viability. Many of their immunomodulatory properties are mediated by specific structural components (e.g., peptidoglycans, teichoic acids) or metabolites that can interact with host cells even after the microorganism is no longer alive (Thoda and Touraki 2023). This realization has given rise to the concept of “paraprobiotics”—inactivated or non‐viable microbial cells or cell fractions that confer a health benefit. Paraprobiotics offer significant advantages over their live counterparts, including a superior safety profile, longer shelf life, and greater ease of standardization and storage, making them highly attractive candidates for clinical development (Nataraj et al. 2020; Lee et al. 2023).

A growing body of evidence indicates that paraprobiotics retain immunomodulatory activity and can attenuate inflammatory responses in several experimental disease models (Teame et al. 2020; Martyniak et al. 2021; Rezaie et al. 2024). Based on their proven anti‐inflammatory properties and their enhanced safety and stability, we hypothesize that paraprobiotics could represent a novel and effective therapeutic strategy for the prevention and treatment of gastric ulcers. We propose that paraprobiotics can protect the gastric mucosa by mitigating destructive inflammatory and oxidative stress responses and, more specifically, by inhibiting the PANoptosis pathway that drives inflammation‐associated cell death.

Therefore, the present study was designed to investigate and compare the gastroprotective effects of the well‐documented probiotic strain *Bifidobacterium animalis* subsp. *lactis* BB‐12 ( *B. lactis* BB‐12) and its paraprobiotic derivative obtained through heat inactivation in a murine model of indomethacin‐induced gastric ulcer. Beyond assessing macroscopic and histological damage, we aimed to elucidate the underlying molecular mechanisms by comprehensively analyzing key markers of oxidative stress and inflammation. Most importantly, this study is the first to investigate whether the protective effects of this paraprobiotic are mediated through the modulation of the PANoptosis pathway by analyzing the expression levels of its core regulatory proteins.

## 2 Materials and Methods

### 2.1 Probiotic Activation and Paraprobiotic Preparation

A lyophilized strain of *B. lactis* BB‐12 was activated in de Man, Rogosa, and Sharpe (MRS) broth at 37°C for 24 h under anaerobic conditions. Following activation, the bacterial culture was harvested by centrifugation at 5000 ***g*** for 10 min at 4°C. The resulting bacterial pellet was washed twice with sterile phosphate‐buffered saline (PBS, pH 7.2) and then resuspended in PBS to a final concentration of 1 × 10^9^ CFU/mL for experimental use. The literature indicates that administering the *B. lactis* BB‐12 strain at a dosage range of 10^8^–10^11^ CFU/day results in resistance to gastrointestinal transit, enhancement of epithelial barrier function, and modulation of the immune system (Collins et al. 2025). In alignment with these findings, the dose employed in the present study is consistent with the effective range reported in the literature. The two post‐inactivation washing steps were applied to standardize the preparation to its cell‐associated structural fraction (peptidoglycan, teichoic and lipoteichoic acids, and surface‐layer proteins), which are recognized as the principal effectors of paraprobiotic bioactivity; the agent tested here therefore represents a washed paraprobiotic (intact non‐viable cells) rather than a soluble postbiotic fraction, and we acknowledge that this deliberate choice may have removed part of the heat‐released soluble components in exchange for improved batch‐to‐batch reproducibility (Lee et al. 2023; Almada et al. 2021).

For paraprobiotic preparation, the activated live *B. lactis* BB‐12 suspension was heat‐inactivated. Specifically, the probiotic suspension, at a concentration of 1 × 10^9^ CFU/mL, was subjected to heat treatment in a water bath at 85°C for 15 min. The suspension was then rapidly cooled on ice, centrifuged, and washed twice with PBS to remove extracellular components. Finally, the inactivated bacterial pellet was resuspended in PBS to a final concentration of 1 × 10^9^ CFU/mL (Almada et al. 2021).

### 2.2 Verification of Probiotic Inactivation by Flow Cytometry

Flow cytometry was performed to confirm that the heat treatment successfully inactivated the bacterial cells and compromised their membrane integrity. A commercial LIVE/DEAD BacLight Bacterial Viability Kit containing SYTO 9 and propidium iodide (PI) dyes (Catalog No.: L34856; Thermo Fisher Scientific, USA) was used to distinguish live and dead cells. Both live probiotic and heat‐treated paraprobiotic samples were incubated with the dye mixture in the dark for 15 min, following the manufacturer's instructions. The detailed method was described previously (Marsak et al. 2026). Flow cytometric analysis was performed using a BD FACSCanto II system (Becton, Dickinson and Company, NJ, USA). Successful probiotic inactivation was confirmed by a shift from the SYTO 9–dominant, PI‐negative profile characteristic of the live‐probiotic suspension to a predominantly PI‐positive (red) profile in the heat‐treated paraprobiotic suspension because the live‐probiotic profile served only to confirm assay validity. The flow cytometric dot plots reported in Figure 1 and Section 3.1 correspond to the paraprobiotic sample.

**FIGURE 1:**
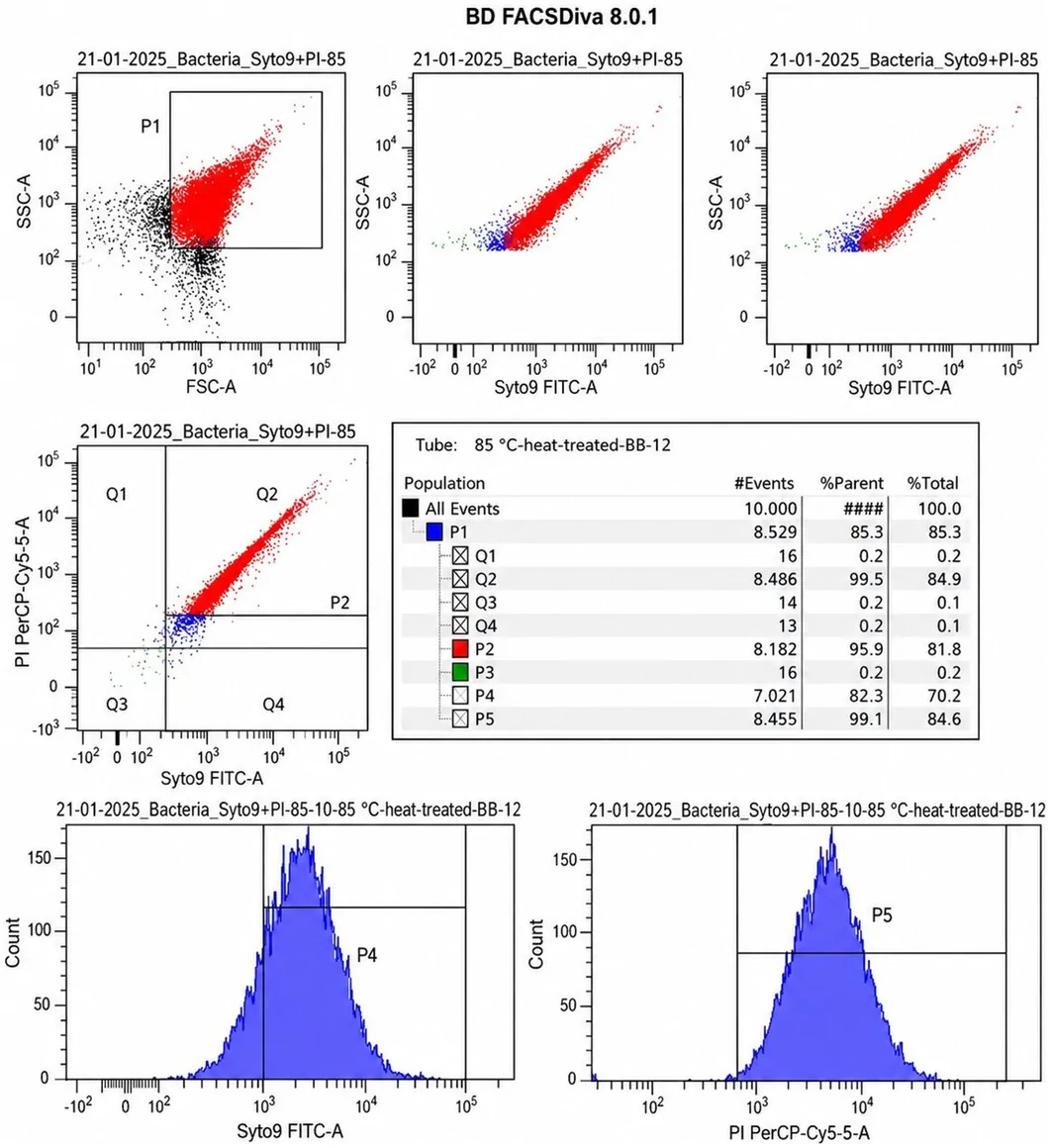
Viability analysis of heat‐treated *B. lactis* BB‐12 population by flow cytometry. Flow cytometry results confirming the efficacy of the heat treatment protocol used in the preparation of paraprobiotics. The forward versus side scatter (FSC vs. SSC) plot shows the P1 gate used to identify the bacterial population (red). The Q2 quadrant represents PI‐positive (dead) cells with compromised membrane integrity, while the Q4 quadrant represents PI‐negative (live) cells with intact membrane integrity. The P4 and P5 histograms show the distribution of SYTO 9 and PI fluorescence intensity, respectively, with the strong signal in P5 confirming that the population is predominantly PI‐positive.

### 2.3 Animals and Ethical Approval

A total of 50 adult male Balb/c mice, weighing between 20 and 26 g, were used in this study. The animals were housed under standard laboratory conditions (22°C ± 2°C, 50%–60% humidity, 12‐h light/dark cycle) with ad libitum access to standard pellet chow and water. All experimental procedures were approved by the Animal Experiments and Ethics Committee of Aydın Adnan Menderes University (Approval No: 64583101/2025/087) and were conducted in accordance with national and international guidelines for animal welfare. Only male mice were used to avoid the confounding influence of the oestrous cycle and fluctuating estrogen levels, which are known to modulate gastric acid secretion, mucosal blood flow, and susceptibility to ulceration, thereby minimizing inter‐individual variability in the ulcer response (Chen et al. 2019). Indomethacin and lansoprazole were dissolved in 5% NaOH, and the control group animals received an equivalent volume of distilled water.

### 2.4 Preparation of Drug Solutions and Dosage Calculation

Indomethacin and lansoprazole solutions were freshly prepared prior to administration. Due to the poor water solubility of these compounds, a specific dilution protocol using 5% NaOH as a stock solvent was utilized to ensure complete dissolution while preventing mucosal injury from the vehicle (Boyacioglu et al. 2016).

#### 2.4.1 Indomethacin Preparation

To achieve the target dose of 25 mg/kg in a standard administration volume of 200 μL (0.2 mL) per mouse (approx. 25 g body weight), a final concentration of 3.125 mg/mL was required. Briefly, 31.25 mg of indomethacin was first dissolved in a minimal volume (0.5 mL) of 5% NaOH to create a concentrated stock. This stock was immediately diluted with 9.5 mL of distilled water to reach a final volume of 10 mL. The indomethacin (25 mg/kg) and lansoprazole (30 mg/kg) doses were selected on the basis of previously validated indomethacin‐induced gastric ulcer models that reproducibly generate mucosal injury and a measurable reference‐drug response (Boyacioglu et al. 2016; Ruiz‐Hurtado et al. 2021).

#### 2.4.2 Lansoprazole Preparation

Similarly, for the 30 mg/kg lansoprazole dose (requiring a final concentration of 3.75 mg/mL), 37.5 mg of lansoprazole was dissolved in 0.5 mL of 5% NaOH stock and subsequently diluted with 9.5 mL of distilled water.

Through this dilution protocol (1:20 ratio), the final concentration of NaOH in the administered vehicle was reduced to 0.25% (v/v). This trace concentration is non‐toxic to the gastric mucosa.

### 2.5 Experimental Design and Ulcer Induction

The gastric ulcer animal model employed was adapted with minor modifications from the protocol described by (Ruiz‐Hurtado et al. 2021). The mice were randomly divided into five groups of 10 animals each (*n* = 10):

- Control Group: Representing the normal (basal) conditions, this group consisted of healthy mice that received only 200 μL of distilled water via gastric gavage and were not subjected to any other treatment.
- Ulcer Group (UG): In this group, acute gastric ulcer was induced by a single administration of indomethacin (25 mg/kg) in a 200 μL volume via gastric gavage. This group served as the positive ulcer control.
- Lansoprazole (LAN) Group: This group served as the reference treatment group. The mice received lansoprazole (30 mg/kg) in a 200 μL volume via oral gastric gavage, 5 min before the administration of indomethacin (25 mg/kg). This short interval was selected to evaluate the immediate topical protective capacity of the drug and to mimic a clinical co‐administration scenario.
- Probiotic (PRO) Group: The mice in this group were pre‐treated orally via gastric gavage with a 200 μL suspension of live *B. lactis* BB‐12 (1 × 10^9^ CFU/mL), 5 min before the administration of indomethacin to ensure the physical presence of the bacterial suspension on the gastric mucosa at the onset of injury.
- Paraprobiotic (PARA) Group: This group was pre‐treated orally via gastric gavage with a 200 μL suspension of heat‐inactivated (paraprobiotic) *B. lactis* BB‐12 (1 × 10^9^ CFU/mL), 5 min before indomethacin administration.

Indomethacin‐induced gastric damage involves a progressive cascade of prostaglandin inhibition and inflammatory infiltration. Therefore, a duration of 6 h was allowed after indomethacin administration to establish the full extent of the lesions (Banerjee et al. 2008). Although the agents were delivered 5 min before indomethacin to guarantee their presence on the mucosal surface at the onset of injury, they remained in contact with the gastric mucosa and the host throughout the entire 6‐h period of lesion development. This window provided sufficient time for the bacterial surface components to engage mucosal pattern‐recognition receptors and for the agents to exert their systemic pharmacological and immunomodulatory effects, thereby mitigating the progression of the ulcers (Lebeer et al. 2010).

Six hours after the final indomethacin administration, all mice were sacrificed under anesthesia via an intraperitoneal injection of ketamine (50 mg/kg) and xylazine (5 mg/kg). Under deep anesthesia, thoracotomy was performed, and intracardiac blood samples were collected. The blood samples were allowed to clot at room temperature before centrifugation to obtain serum. Immediately following blood collection, the stomachs were rapidly excised, rinsed with cold PBS to remove their contents, and opened for macroscopic evaluation (Boyacioglu et al. 2016).

### 2.6 Macroscopic Evaluation of Gastric Tissue

The stomachs were photographed alongside a reference scale. The area of ulcerative lesions (hyperemia and hemorrhagic erosions) in the gastric mucosa was measured in square millimeters (mm^2^) using LabSense image analysis software (Olympus, Japan) (Adem and Selim 2025). For each stomach, the ulcer area (%) was defined as the proportion of the total gastric mucosal surface occupied by ulcerative lesions, and this value is the parameter reported in Table 1. The protective effect of the treatments was calculated as the percentage of ulcer inhibition (%І) using the following formula:

$$\text{Inhibition Percentage}\;\left(І\%\right)=\left[\left(\mathrm{USc}—\mathrm{USt}\right)/\mathrm{USc}\right]\times 100$$

where USc is the mean ulcer surface area in the ulcer control group and USt is the mean ulcer surface area in the treated group.

**TABLE 1:** Effects of indomethacin, lansoprazole, *B. lactis* BB‐12 probiotic, and *B.* lactis BB‐12 paraprobiotic on indomethacin‐induced gastric ulcers in mice: Ulcer area (%) and damage score (*n* = 10 biological replicates in each group).

Groups	Drugs	Dose	Ulcer area (%)	Ulcer Inhibition (%)	Macroscopic damage score
Control	Control	Distilled water	0.0 ± 0.0^a^	—	0.0 ± 0.0^a^
UG	Indomethacin	25 mg/kg	52.54 ± 3.21^b^	—	3.98 ± 0.22^b^
LAN	Lansoprazole	30 mg/kg	15.11 ± 2.33^c^	71.2	2.08 ± 0.13^c^
PRO	*B. lactis* BB‐12 Probiotic	1 × 10^9^ CFU/mL	21.57 ± 1.27^c^	58.9	1.87 ± 0.21^c^
PARA	*B. lactis* BB‐12 Paraprobiotic	1 × 10^9^ CFU/mL	17.34 ± 2.09^c^	67.0	1.02 ± 0.10^d^
			***		***

*Note:* Data are expressed as the mean ± standard error of the mean (SEM), *n* = 10 animals per group. For each parameter, the five groups were compared by one‐way analysis of variance (ANOVA) followed by Tukey's post hoc test. The lowercase superscript letters (a–d) next to the values are a compact‐letter display of these pairwise comparisons: within a given column, groups that do not share a common letter differ significantly (*p* < 0.05), whereas groups sharing a letter are not significantly different. The three asterisks (***) in the bottom row of each parameter column are not a data value; they denote the overall (omnibus) effect of group on that parameter, that is, the significance of the corresponding one‐way ANOVA across all groups (***p < 0.001). Accordingly, *** indicates that the between‐group differences for that parameter are highly significant overall, while the superscript letters identify which specific groups differ. Ulcer inhibition percentage was calculated as (USc minus USt) divided by USc, multiplied by 100, where USc is the mean ulcer area of the UG and USt is the mean ulcer area of the treated group; because the UG itself defines the reference value, no inhibition percentage is reported for the Control and UG rows.

Abbreviations: Control, healthy control group; LAN, lansoprazole; PARA, paraprobiotic; PRO, live probiotic; UG, ulcer group (indomethacin only).

Additionally, gastric damage was semi‐quantitatively assessed using a 0–4 scoring system: 0: normal mucosa; 0.5: hyperemia only; 1: one or two small lesions; 2: severe lesions; 3: very severe lesions; 4: mucosa completely covered with lesions (Boyacioglu et al. 2016). The scoring was performed by three independent investigators who were blinded to the treatment assignments.

### 2.7 Histopathological Examination

Following macroscopic examination, stomach tissue samples were fixed in 10% buffered formalin solution for 24 h. The fixed tissues were then processed through routine histological procedures (dehydration, clearing, paraffin infiltration) and embedded in paraffin blocks. Sections of 5 μm thickness were cut, stained with hematoxylin and eosin (H&E), and examined under a light microscope (Olympus, Japan). Microscopic damage was evaluated using a semi‐quantitative scoring system adapted from Laine and Weinstein (Laine and Weinstein 1988). Four histopathological parameters, namely occluded blood vessels, hemorrhage, inflammatory cell infiltration, and edema, were each independently graded on a 0–4 scale (0: absent; 4: most severe), and the total damage index for each animal was calculated as the average of the four parameter scores (maximum possible score: 4.0).

### 2.8 Biochemical Analyses

#### 2.8.1 Serum Cytokine Analysis

Collected blood samples were centrifuged at 3000 rpm for 10 min to separate the serum. The levels of pro‐inflammatory (TNF‐α, IL‐6) and anti‐inflammatory (IL‐10) cytokines in the serum were measured using commercial ELISA (Enzyme‐Linked Immunosorbent Assay) kits according to the manufacturer's instructions.

#### 2.8.2 Tissue Homogenate Preparation and Analyses

Stomach tissue samples were weighed after being rinsed with ice‐cold PBS. The tissues were homogenized in a 10% (1/10 w/v) solution of 150 mM phosphate buffer (pH 7.4) in an ice bath using a homogenizer (IKA Overhead Stirrer, Germany). The homogenate was centrifuged at 7000 ***g*** for 10 min at 4°C, and the supernatants were stored at −80°C until analysis. The total protein concentration in the supernatants was measured by the Biuret method using commercial kits (Archem Diagnostic, Turkey).

#### 2.8.3 Antioxidant Enzyme Activities and Glutathione Levels

SOD activity was measured at 560 nm based on the inhibition of nitro blue tetrazolium reduction by the xanthine/xanthine oxidase system and expressed as U/mg of tissue protein (Sun et al. 1988). CAT activity was determined by monitoring the decomposition of H_2_O_2_ at 240 nm and expressed as k/mg of tissue protein (Boyacioglu et al. 2016). The level of reduced GSH was measured spectrophotometrically at 412 nm using the Tietze method and expressed as nmol/g of tissue protein (Tietze 1969).

#### 2.8.4 Assessment of Lipid Peroxidation and MPO Activity

Lipid peroxidation was determined by measuring malondialdehyde (MDA) levels using the thiobarbituric acid reactive substances (TBARS) assay, as described previously (Yoshioka et al. 1979). To evaluate neutrophil accumulation, MPO activity was measured spectrophotometrically at 460 nm, with the results expressed as nmol/min/mg tissue protein (Bradley et al. 1982). Each assay was conducted in duplicate, and the results were averaged.

### 2.9 Western Blot Analysis

Western blot analysis was performed to determine the expression levels of proteins involved in the PANoptosis pathway. Proteins extracted from tissue homogenates (30 μg per well) were separated by 10%–12% sodium dodecyl sulfate‐polyacrylamide gel electrophoresis (SDS‐PAGE) and transferred electrophoretically to polyvinylidene difluoride (PVDF) membranes. The membranes were blocked for 1 h at room temperature with 5% non‐fat dry milk in Tris‐buffered saline with Tween 20 (TBST). The membranes were then incubated overnight at 4°C with the following primary antibodies (all supplied by Cell Signaling Technology, Danvers, MA, USA): MLKL (#37705), Phospho‐MLKL (Ser345, #37333), Caspase‐3 (#14220), Phospho‐RIPK3 (Thr231/Ser232, #91702), Gasdermin D (#39754), Cleaved Gasdermin D (Asp276, #10137), IL‐1β (#12507), Cleaved‐IL‐1β (Asp117, #63124), RIPK3 (#95702), and β‐actin as a loading control. After washing, the membranes were incubated for 1 h at room temperature with the appropriate HRP‐linked secondary antibody (Anti‐rabbit IgG, HRP‐linked Antibody #7074; Cell Signaling Technology, Danvers, MA, USA). Protein bands were visualized using an enhanced chemiluminescence (ECL) detection kit and subjected to densitometric analysis (Adem and Selim 2025).

### 2.10 Statistical Analysis

The normality of all data was checked using the Shapiro–Wilk test, and the homogeneity of variances was assessed using Levene's test. For normally distributed data, comparisons between groups were performed using one‐way analysis of variance (ANOVA) followed by Tukey's post hoc test for all pairwise comparisons. For data that were not normally distributed, the Kruskal‐Wallis test followed by the Mann–Whitney U test with Bonferroni correction was used. All analyses were conducted using GraphPad Prism 9 software (Version 9.0). A *p*‐value of < 0.05 was considered statistically significant. Data are expressed as mean ± standard error of the mean (SEM).

## 3 Results

### 3.1 Verification of Probiotic Inactivation by Flow Cytometry

The successful inactivation of the *B. lactis* BB‐12 strain for its use as a paraprobiotic was confirmed by flow cytometry. In this analysis, the heat‐treated (paraprobiotic) suspension was evaluated for viability and membrane integrity using the nucleic acid dyes SYTO 9 and propidium iodide (PI); the corresponding dot plots and histograms are presented in Figure 1. Bacterial events were first gated on P1 and then on P2, the singlet bacterial population, which comprised 95.9% of the P1 gate. Within the P2 population, a clear majority of the cells were concentrated in the Q2 quadrant (SYTO 9 positive/PI positive), which represents dead cells with compromised membrane integrity; quantitative analysis showed that 99.5% of the P2 population fell within this quadrant. Conversely, a negligible fraction (< 0.2%) of the population was detected in the Q4 quadrant (SYTO 9 positive/PI negative), which represents viable cells with intact membranes. These findings were further supported by the histograms generated for SYTO 9 (P4) and PI (P5): the P5 histogram confirmed the inactivation, showing that nearly the entire population emitted a strong PI signal. The untreated live‐probiotic suspension was analyzed in parallel using the same gating strategy and showed the SYTO 9‐dominant, PI‐negative profile characteristic of an intact membrane, consistent with our previously validated protocol (Marsak et al. 2026); as this profile served only to confirm assay validity, only the paraprobiotic dot plots are shown in Figure 1. Collectively, these data indicate that the applied heat treatment protocol inactivated *B. lactis* BB‐12 with high efficiency (over 99%) and that the paraprobiotic suspension used in this study was devoid of viable cells.

### 3.2 Protective Effects of Probiotics and Paraprobiotics on the Gastric Mucosa

The macroscopic damage to the gastric mucosa following indomethacin administration and the protective effects of the treatment groups were quantitatively evaluated based on ulcer area and damage score. The results are summarized in Table 1, and representative macroscopic images are shown in Figure 2.

**FIGURE 2:**
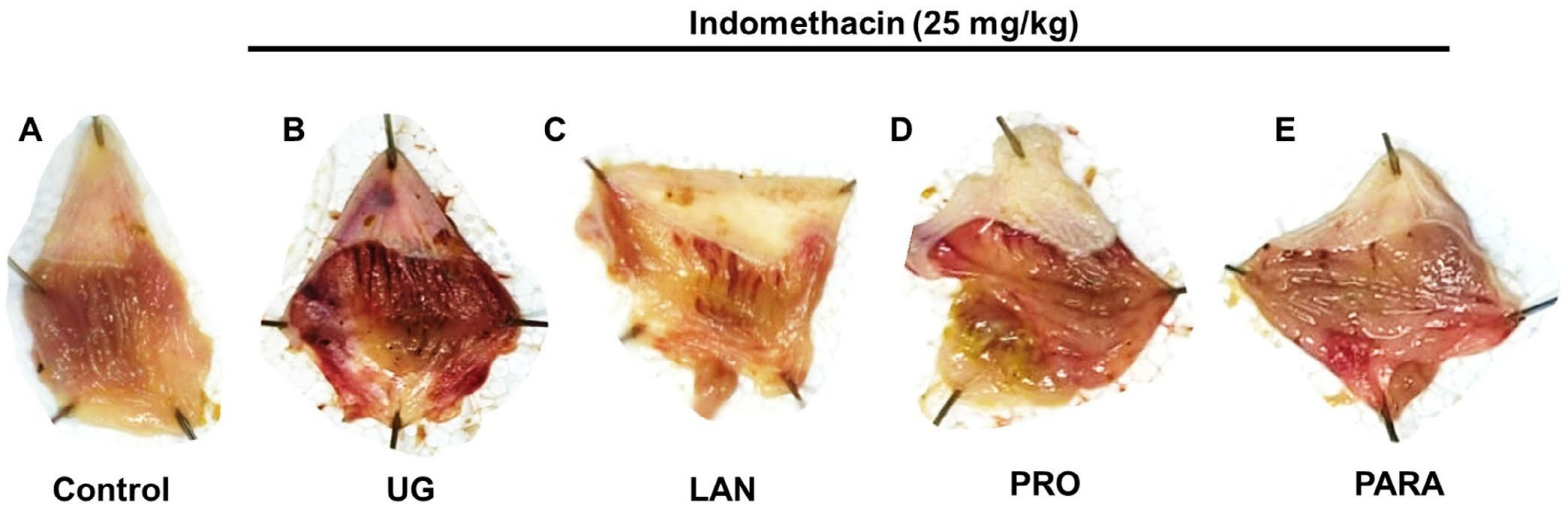
Representative macroscopic images of stomach tissues from all experimental groups (*n* = 10 biological replicates per group). Representative macroscopic images of stomach tissues from all experimental groups. (A) Control Group: Healthy and normal gastric mucosa with no lesions. (B) Ulcer Group (UG): Widespread and severe hemorrhagic lesions, linear ulcers, and edema in the gastric mucosa following indomethacin administration. (C) Lansoprazole Group (LAN): Pre‐treatment with the reference drug lansoprazole shows a marked reduction in the severity and extent of lesions compared to the ulcer group. (D) Probiotic Group (PRO): Pre‐treatment with live *B. lactis* BB‐12 significantly prevented indomethacin‐induced gastric damage and largely preserved mucosal integrity. (E) Paraprobiotic Group (PARA): Pre‐treatment with heat‐inactivated paraprobiotic *B. lactis* BB‐12 exhibited a potent gastroprotective effect similar to the probiotic group, markedly suppressing the formation of hemorrhagic lesions.

The gastric mucosa of the control group mice exhibited a normal and healthy appearance, with no signs of damage, hyperemia, or hemorrhagic lesions (Figure 2A). As expected, the ulcer area and macroscopic damage score for this group were recorded as zero (Table 1). In contrast, a single dose of indomethacin induced severe and widespread hemorrhagic lesions, linear ulcers, and edema in the gastric mucosa of the UG (Figure 2B). In this group, the ulcer area covered 52.54% ± 3.21% of the stomach surface, and the macroscopic damage score reached a high value of 3.98 ± 0.22. These values confirm that statistically significant damage occurred compared to the control group (*p* < 0.001). Pre‐treatment with the reference drug, lansoprazole, provided significant protection against the gastric damage caused by indomethacin. Lansoprazole administration reduced the ulcer area to 15.11% ± 2.33% and the damage score to 2.08 ± 0.13, demonstrating a statistically significant gastroprotective effect compared to the UG (*p* < 0.001) (Figure 2C). Similarly, treatment with the live *B. lactis* BB‐12 probiotic (PRO) also exerted a strong protective effect. Probiotic administration significantly reduced the ulcer area (21.57% ± 1.27%) and damage score (1.87 ± 0.21) compared to the UG (*p* < 0.001) (Figure 2D).

To further characterize the macroscopic protection, the ulcer inhibition percentage was calculated relative to the UG (Table 1). Lansoprazole achieved the greatest inhibition (71.2%), followed by the paraprobiotic (67.0%) and the live probiotic (58.9%); however, the ulcer areas of the LAN, PRO, and PARA groups did not differ significantly from one another (*p* > 0.05), indicating comparable macroscopic protection among the three treatments. A clear distinction emerged instead for the macroscopic damage score, where paraprobiotic administration lowered the score to a remarkably low level of 1.02 ± 0.10. This damage score was statistically significantly lower not only than the UG but also than the lansoprazole and probiotic groups (*p* < 0.05). These results indicate that while the three treatments provide comparable protection against ulcer area formation, the paraprobiotic confers the most potent protection specifically at the level of macroscopic damage severity.

### 3.3 Effects of Treatments on Histopathological Changes

A histopathological examination of stomach tissues was performed using Hematoxylin and Eosin (H&E) staining to support the macroscopic findings and evaluate the gastroprotective effects of the treatments at the cellular level. The observed microscopic changes are shown in Figure 3, and the quantitative scoring results, including parameters such as occluded blood vessels, hemorrhage, inflammatory cell infiltration, and edema, are summarized in Table 2.

**FIGURE 3:**
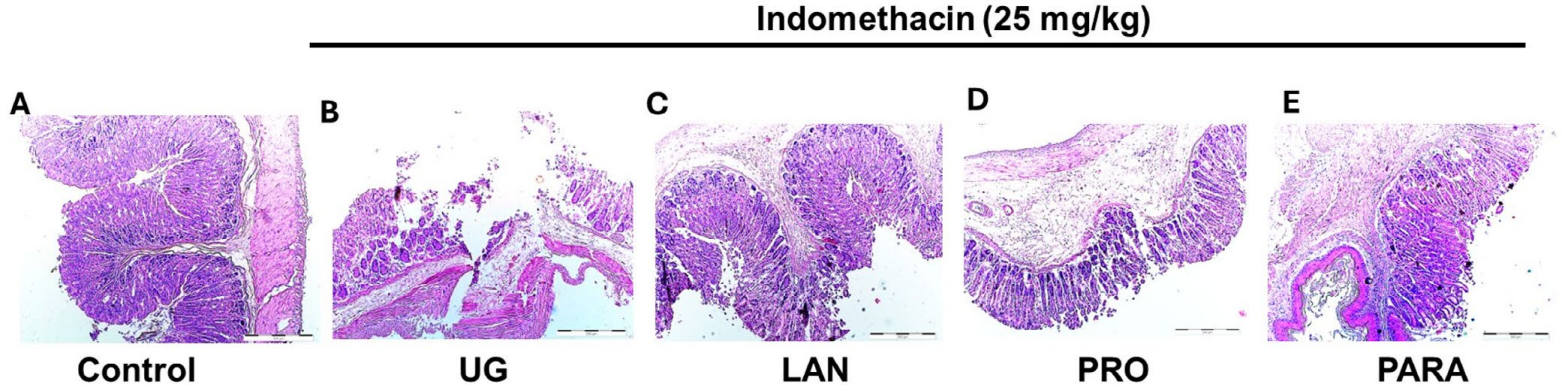
Representative microscopic images of Hematoxylin and Eosin (H&E) stained stomach tissues from different treatment groups in the indomethacin‐induced gastric ulcer model (*n* = 10 biological replicates per group). Microscopic images of Hematoxylin and Eosin (H&E) stained stomach tissues from different treatment groups in the indomethacin‐induced gastric ulcer model. (A) Control group shows normal and healthy gastric mucosal structure. (B) Ulcer Group (UG) exhibits severe mucosal necrosis, deep ulceration, submucosal edema, and massive inflammatory cell infiltration. (C) Lansoprazole (LAN) group shows reduced mucosal damage compared to the ulcer group. (D) Probiotic (PRO) group displays a potent protective effect with largely preserved mucosal structure. (E) Paraprobiotic (PARA) group shows a marked gastroprotective effect and preserved tissue integrity, similar to the probiotic group. (Scale bar = 200 μm).

**TABLE 2:** Scoring of histopathological changes in different treatment groups (*n* = 10 biological replicates/group).

Group	Occluded blood vessels	Hemorrhage	Inflammatory cell infiltration	Edema	Total damage index
Control	0.00 ± 0.00^a^	0.00 ± 0.00^a^	0.00 ± 0.00^a^	0.00 ± 0.00^a^	0.00 ± 0.00^a^
UG	3.91 ± 0.32^b^	4.00 ± 0.23^b^	4.00 ± 0.22^b^	3.85 ± 0.31^b^	3.95 ± 0.22^b^
LAN	2.59 ± 0.41^c^	2.88 ± 0.34^c^	2.29 ± 0.34^c^	2.00 ± 0.22^c^	2.49 ± 0.31^c^
PRO	1.22 ± 0.23^d^	1.00 ± 0.20^d^	1.37 ± 0.22^d^	1.17 ± 0.20^d^	1.27 ± 0.23^d^
PARA	1.16 ± 0.20^d^	0.91 ± 0.21^d^	0.93 ± 0.23^d^	1.05 ± 0.10^d^	0.95 ± 0.20^d^

*Note:* Histopathological evaluation was performed on hematoxylin–eosin‐stained gastric sections based on four parameters: (1) occluded blood vessels (0: absent; 4: widespread occlusion), (2) hemorrhage (0: absent; 4: severe hemorrhage), (3) inflammatory cell infiltration (0: absent; 4: dense neutrophil/mononuclear infiltration), and (4) edema (0: absent; 4: prominent submucosal edema), following the semi‐quantitative grading scheme of Laine and Weinstein (Laine and Weinstein 1988). The total damage index was calculated as the average of the four parameters (maximum 4.0). Data are expressed as mean ± SEM (*n* = 10 animals per group) and were compared among groups by one‐way ANOVA followed by Tukey's post hoc test. Within each column, means not sharing a common superscript letter (a–d) differ significantly (*p* < 0.05); groups sharing a letter are not significantly different. Group abbreviations are as defined in Table 1.

The stomach tissue of mice in the control group displayed a normal and healthy histological structure, characterized by regular gastric pits and glandular structures (Figure 3A). The integrity of the surface epithelium was complete, and no pathological findings were observed in the submucosa. All histopathological scores for this group were recorded as zero, as expected (Table 2). In contrast, severe and widespread histopathological damage was observed in the indomethacin‐treated UG (Figure 3B). Mucosal integrity was completely disrupted, with deep foci of necrosis and erosion in the surface epithelium and glandular layers. Additionally, massive inflammatory cell infiltration, primarily neutrophils, along with severe edema, hemorrhage, and vascular occlusion, was prominent in the submucosa. This severe damage was confirmed by the high scores obtained by the UG for occluded blood vessels (3.91 ± 0.32), hemorrhage (4.00 ± 0.23), inflammatory cell infiltration (4.00 ± 0.22), and edema (3.85 ± 0.31), with a total damage index of 3.95 ± 0.22 (*p* < 0.05 vs. control).

Pre‐treatment with lansoprazole, probiotics, and paraprobiotics significantly attenuated the histopathological damage caused by indomethacin. The reference drug, lansoprazole, provided a statistically significant reduction in all damage parameters and the total damage index (2.49 ± 0.31) compared to the UG (*p* < 0.05 vs. UG). Notably, both the PRO and PARA groups demonstrated a more potent protective effect than lansoprazole. The total damage index decreased to 1.27 ± 0.23 in the PRO group and 0.95 ± 0.20 in the PARA group. The total damage indices of these two groups were statistically significantly lower not only than the UG (*p* < 0.05 vs. UG) but also than the LAN group (*p* < 0.05 vs. LAN). As shown in Figure 3D,E, the mucosal structure in these groups was largely preserved, epithelial integrity was maintained, and submucosal edema and inflammatory cell infiltration were minimal. These microscopic findings corroborate macroscopic evaluations and demonstrate that paraprobiotics administration is highly effective in preventing the histological signs of indomethacin‐induced gastric damage.

### 3.4 Effects of Probiotics and Paraprobiotics on Oxidative Stress Markers and Myeloperoxidase Activity

The activities of endogenous antioxidant enzymes (SOD, CAT), the level of reduced GSH, the level of MDA as an indicator of lipid peroxidation, and the activity of MPO as a marker of neutrophil infiltration were measured in stomach tissue homogenates to evaluate the role of oxidative stress in the pathogenesis of indomethacin‐induced gastric ulcer and the antioxidant potential of the treatments (Figure 4).

**FIGURE 4:**
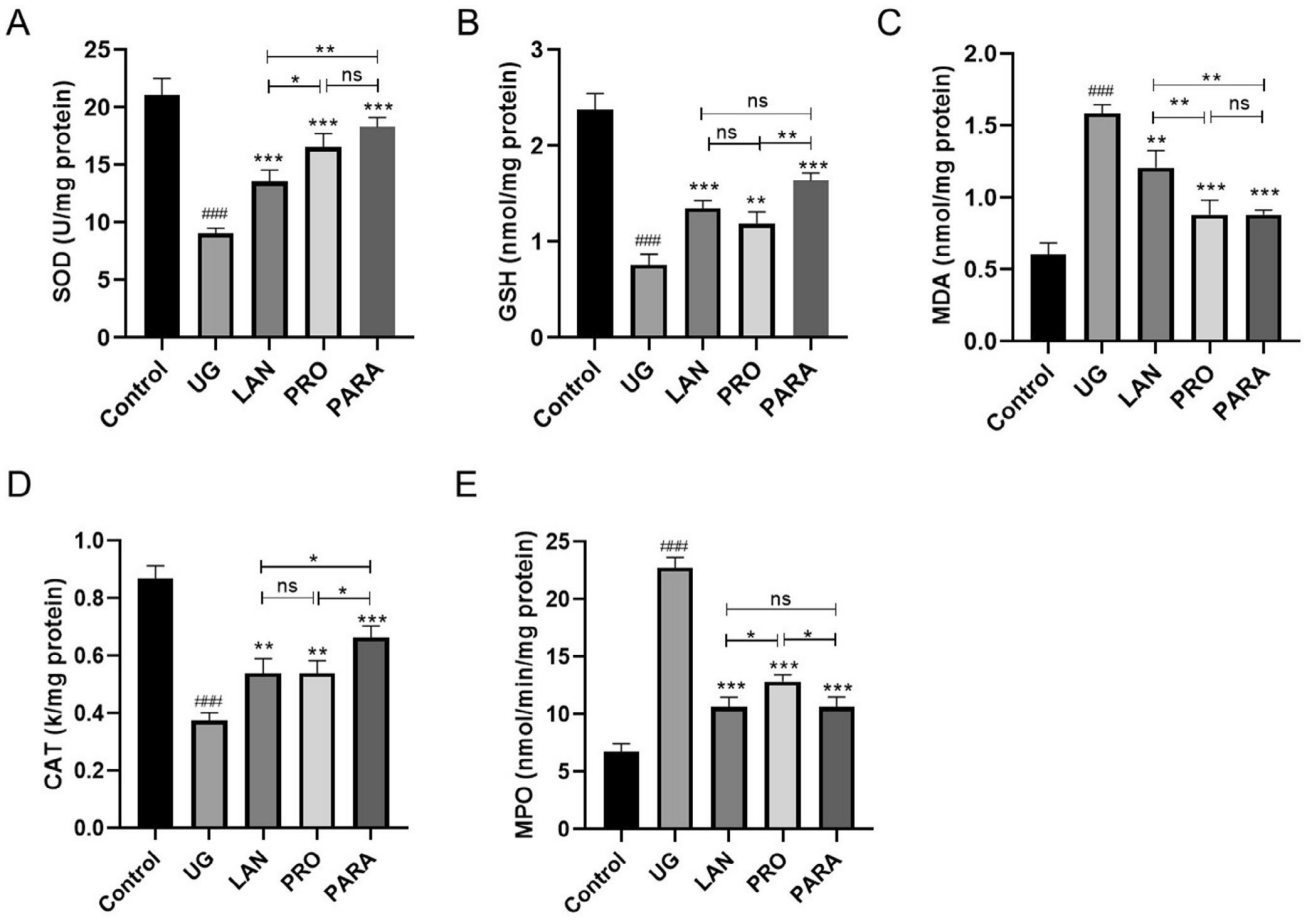
Effects of probiotics and paraprobiotics on oxidative stress and inflammation markers in stomach tissue. Measured levels of (A) Superoxide dismutase (SOD) activity, (B) reduced Glutathione (GSH), (C) Malondialdehyde (MDA), (D) Catalase (CAT) activity, and (E) Myeloperoxidase (MPO) activity in stomach tissue homogenates. Data are presented as mean ± standard error of the mean (SEM) (*n* = 10 biological replicates per group). Statistical analysis was performed using one‐way ANOVA followed by Tukey's post hoc test. ^###^ *p* < 0.001, vs. Control group. *p < 0.05, **p < 0.01, ***p < 0.001, vs. Ulcer group (UG) or as indicated between groups. ns, not significant.

Compared to the control group, the antioxidant defense system was significantly compromised in the indomethacin‐treated UG. The levels of SOD (Figure 4A), GSH (Figure 4B), and CAT (Figure 4D) were statistically significantly decreased in the UG (^###^ *p* < 0.001). Conversely, pre‐treatment with lansoprazole, probiotics, and paraprobiotics significantly restored the levels of these antioxidant markers compared to the UG (*p* < 0.05, *p* < 0.01, or *p* < 0.001). Paraprobiotics administration, in particular, exerted the most potent protective effect by elevating SOD, GSH, and CAT levels to near‐control group levels. As an indicator of oxidative damage, the MDA level (Figure 4C) was sharply increased in the UG compared to the control group (^###^ *p* < 0.001). All treatment groups significantly suppressed this increase (*p* < 0.01 or *p* < 0.001). In preventing lipid peroxidation, both the probiotic and the paraprobiotic groups reached significantly lower MDA levels than the reference drug lansoprazole (LAN vs. PRO and LAN vs. PARA, *p* < 0.01; Figure 4C), whereas the difference between the probiotic and paraprobiotic groups was not significant (ns).

Myeloperoxidase activity (Figure 4E), an indicator of inflammation and neutrophil accumulation, was significantly elevated in the ulcer group compared to the control group (^###^ *p* < 0.001). Lansoprazole, probiotic, and paraprobiotics administrations successfully suppressed the increased MPO activity (*p* < 0.001), indicating that they reduced indomethacin‐induced neutrophil infiltration. There was no statistically significant difference in the reduction of MPO activity between the LAN and PARA treatment groups (*p* > 0.05). These findings reveal that probiotics and paraprobiotics exert a gastroprotective effect by mitigating oxidative stress through the reinforcement of the endogenous antioxidant defense system and the suppression of neutrophil‐mediated inflammation.

### 3.5 Effects of Probiotics and Paraprobiotics on the Systemic Inflammatory Response

The serum levels of pro‐inflammatory cytokines (TNF‐α, IL‐6) and the anti‐inflammatory cytokine (IL‐10) were measured to evaluate the immunomodulatory effects of the treatments on the systemic inflammatory response (Figure 5). As expected, the serum levels of the pro‐inflammatory cytokines TNF‐α and IL‐6 were statistically significantly increased in the indomethacin‐treated UG compared to the control group (^###^ *p* < 0.001). All pre‐treatments with lansoprazole, probiotics, and paraprobiotics significantly suppressed this indomethacin‐triggered cytokine surge (*p* < 0.001). Notably, the PARA group exhibited the most potent anti‐inflammatory effect, reducing TNF‐α and IL‐6 levels more significantly than both the reference drug lansoprazole and the live probiotic (*p* < 0.01 and *p* < 0.05, respectively).

**FIGURE 5:**
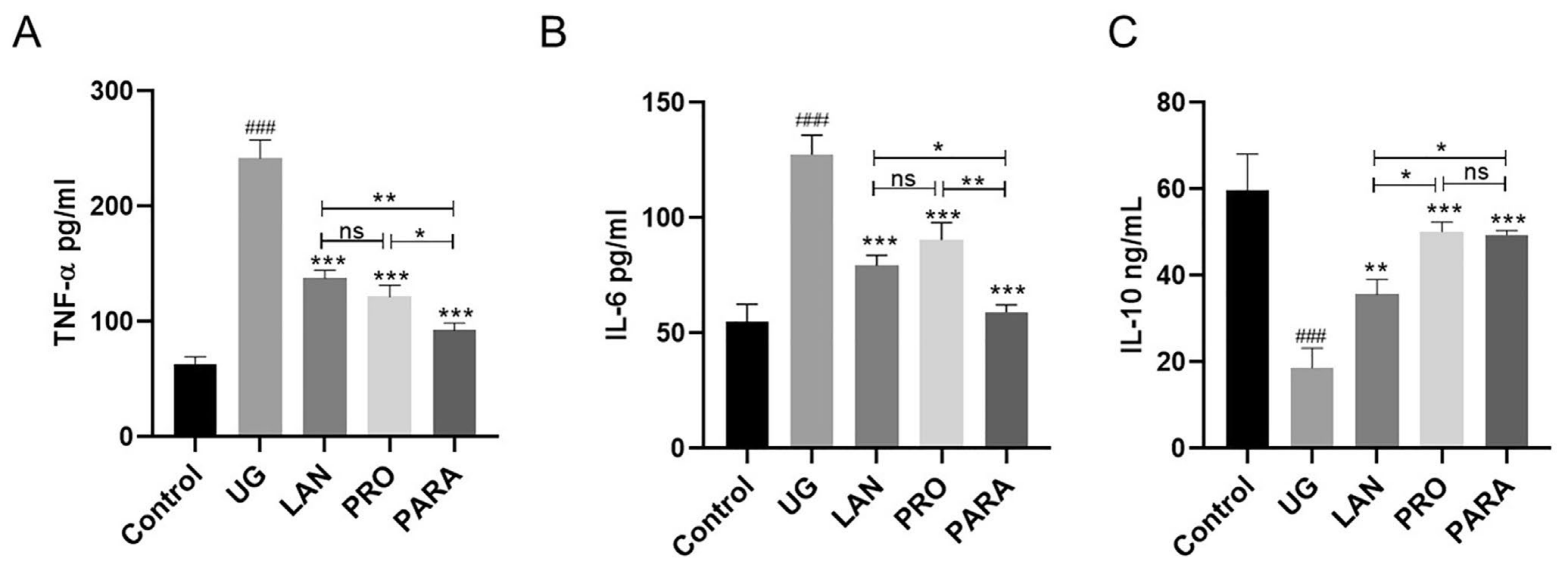
Effects of treatments on serum cytokine levels. Measured serum levels of pro‐inflammatory cytokines (A) TNF‐α, (B) IL‐6, and the anti‐inflammatory cytokine (C) IL‐10 in all experimental groups. Data are presented as mean ± standard error of the mean (SEM) (*n* = 10 biological replicates per group). Statistical analysis was performed using one‐way ANOVA followed by Tukey's post hoc test. ^###^ *p* < 0.001, vs. Control group. *p < 0.05, **p < 0.01, ***p < 0.001, vs. Ulcer group (UG) or as indicated between groups. ns, not significant.

Conversely, the level of the anti‐inflammatory cytokine IL‐10 was significantly decreased in the ulcer group compared to the control group (^###^ *p* < 0.001). All applied treatments succeeded in restoring the suppressed IL‐10 levels. While lansoprazole administration partially restored this level (**p < 0.01), both probiotic and paraprobiotics treatments demonstrated a significantly more potent effect, bringing IL‐10 levels back to the normal levels observed in the healthy control group (***p < 0.001). There was no significant difference in IL‐10 restoration between the probiotic and PARA groups (*p* > 0.05). These results indicate that probiotic and, particularly, paraprobiotics administrations rebalance the systemic cytokine profile by suppressing pro‐inflammatory mediators while enhancing anti‐inflammatory responses, thereby exerting a potent immunomodulatory effect.

### 3.6 Inhibitory Effect of Probiotics and Paraprobiotics on the Pyroptosis Pathway

The expression levels of key proteins involved in the pyroptosis pathway, a crucial component of PANoptosis, were investigated by Western blot analysis to elucidate the molecular mechanisms underlying the gastroprotective effects of the treatments (Figure 6). As shown by the representative blots and densitometric analysis in Figure 6A, the pyroptosis pathway was significantly activated in the indomethacin‐treated UG compared to the control group. The expression of NLRP3, the main component of the NLRP3 inflammasome (Figure 6B), and the subsequent conversion of caspase‐1 to its active, cleaved form (c‐caspase‐1) (Figure 6C) were significantly increased in the UG (^###^ *p* < 0.001). This activation also significantly elevated the conversion of the caspase‐1 substrates pro‐IL‐1β to its mature, cleaved form (c‐IL‐1β) (Figure 6D) and Gasdermin D (GSDMD) to its pore‐forming active fragment (c‐GSDMD) (Figure 6E) (^###^ *p* < 0.001).

**FIGURE 6:**
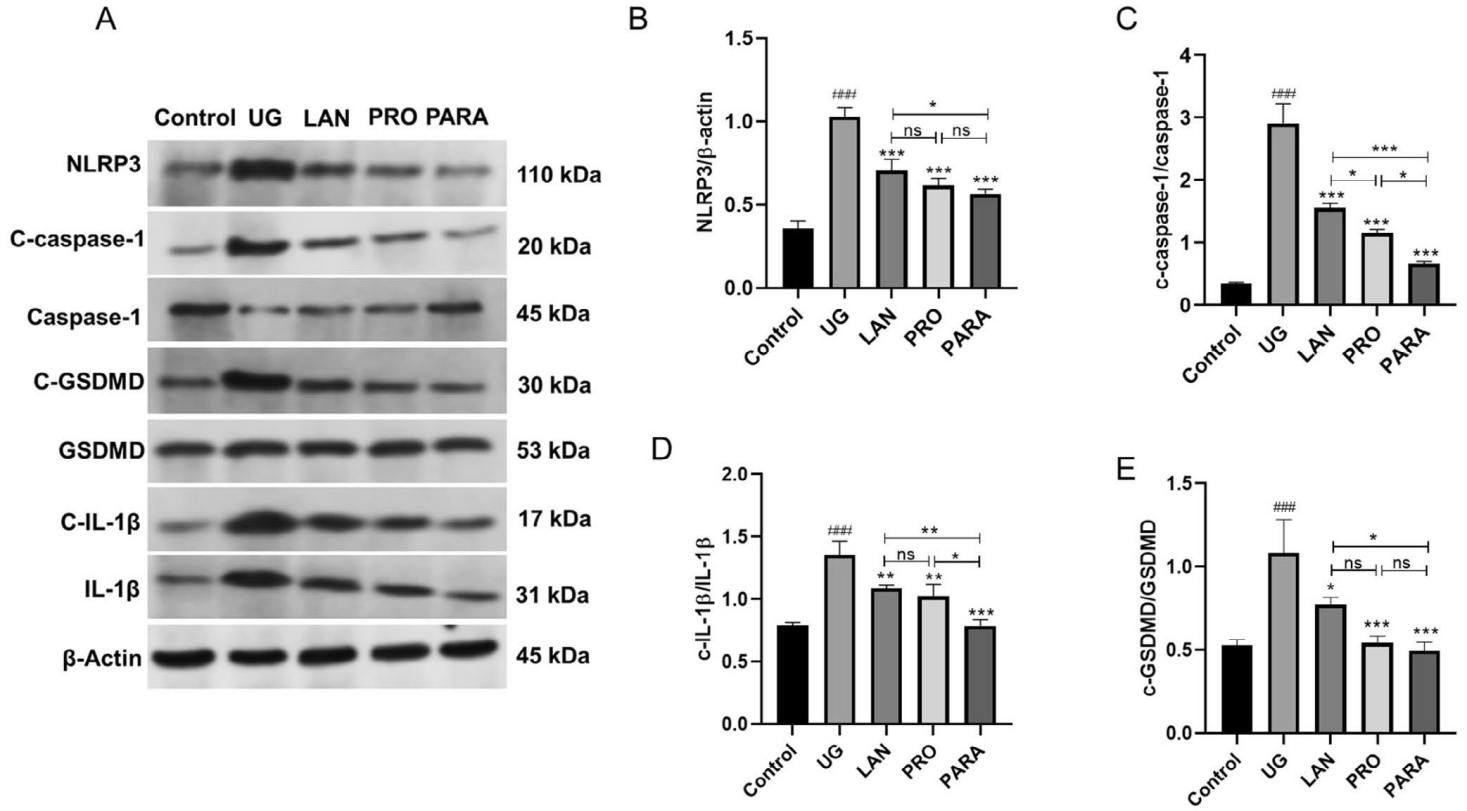
Effects of probiotics and paraprobiotics on pyroptosis pathway proteins in stomach tissue. (A) Representative Western blot bands for NLRP3, Caspase‐1, cleaved Caspase‐1 (C‐caspase‐1), Gasdermin D (GSDMD), cleaved GSDMD (C‐GSDMD), IL‐1β, cleaved IL‐1β (C‐IL‐1β), and β‐Actin as a loading control. (B–E) Densitometric analysis results for the normalized density of NLRP3 to β‐Actin, the ratio of C‐caspase‐1/Caspase‐1, the ratio of C‐IL‐1β/IL‐1β, and the ratio of C‐GSDMD/GSDMD, respectively. Data are presented as mean ± standard error of the mean (SEM) (*n* = 3 biological replicates per group). Statistical analysis was performed using one‐way ANOVA followed by Tukey's post hoc test. ^###^ *p* < 0.001, vs. Control group. *p < 0.05, **p < 0.01, ***p < 0.001, vs. Ulcer group (UG) or as indicated between groups. ns, not significant.

In contrast, pre‐treatment with lansoprazole, probiotic, and paraprobiotics significantly suppressed this indomethacin‐triggered pyroptotic activation. All three treatment groups significantly reduced the expression of NLRP3 and the levels of the cleaved forms of caspase‐1, GSDMD, and IL‐1β compared to the UG (*p* < 0.05, *p* < 0.01, or *p* < 0.001). In comparison between treatments, both probiotic and paraprobiotics administrations were found to be more effective than the reference drug lansoprazole in inhibiting the pyroptosis pathway. Notably, the PARA group reduced the levels of cleaved caspase‐1 and cleaved IL‐1β to a significantly greater extent than the live PRO group (*p* < 0.05 for both comparisons; Figure 6C,D). These molecular findings suggest that the gastroprotective effects of probiotics, and particularly paraprobiotics, are mediated, at least in part, through their potent inhibition of the NLRP3 inflammasome‐mediated pyroptotic cell death pathway.

### 3.7 Regulatory Effects of Probiotics and Paraprobiotics on the Apoptosis Pathway

The expression levels of key apoptosis‐related proteins were examined by Western blot analysis to investigate the role of apoptosis, another key component of the PANoptosis pathway, in the pathogenesis of gastric ulcer and the effects of the treatments on this pathway (Figure 7). The analysis revealed that the apoptosis pathway was potently activated in the indomethacin‐treated UG. Compared to the control group, the expression of the PANoptosis sensor ZBP1 (Figure 7C) and the conversion of the initiator caspase, caspase‐8, to its active, cleaved form (c‐caspase‐8) (Figure 7B) were significantly increased in the UG (^###^ *p* < 0.001). These initiator signals also triggered the activation of the executioner caspase, caspase‐3, leading to a marked increase in cleaved caspase‐3 (c‐caspase‐3) levels (Figure 7D) (^###^ *p* < 0.001). Furthermore, the levels of cleaved Gasdermin E (c‐GSDME), the active form of a caspase‐3 substrate that can trigger secondary necrosis upon cleavage, were also significantly elevated in the UG (Figure 7E) (^###^ *p* < 0.001).

**FIGURE 7:**
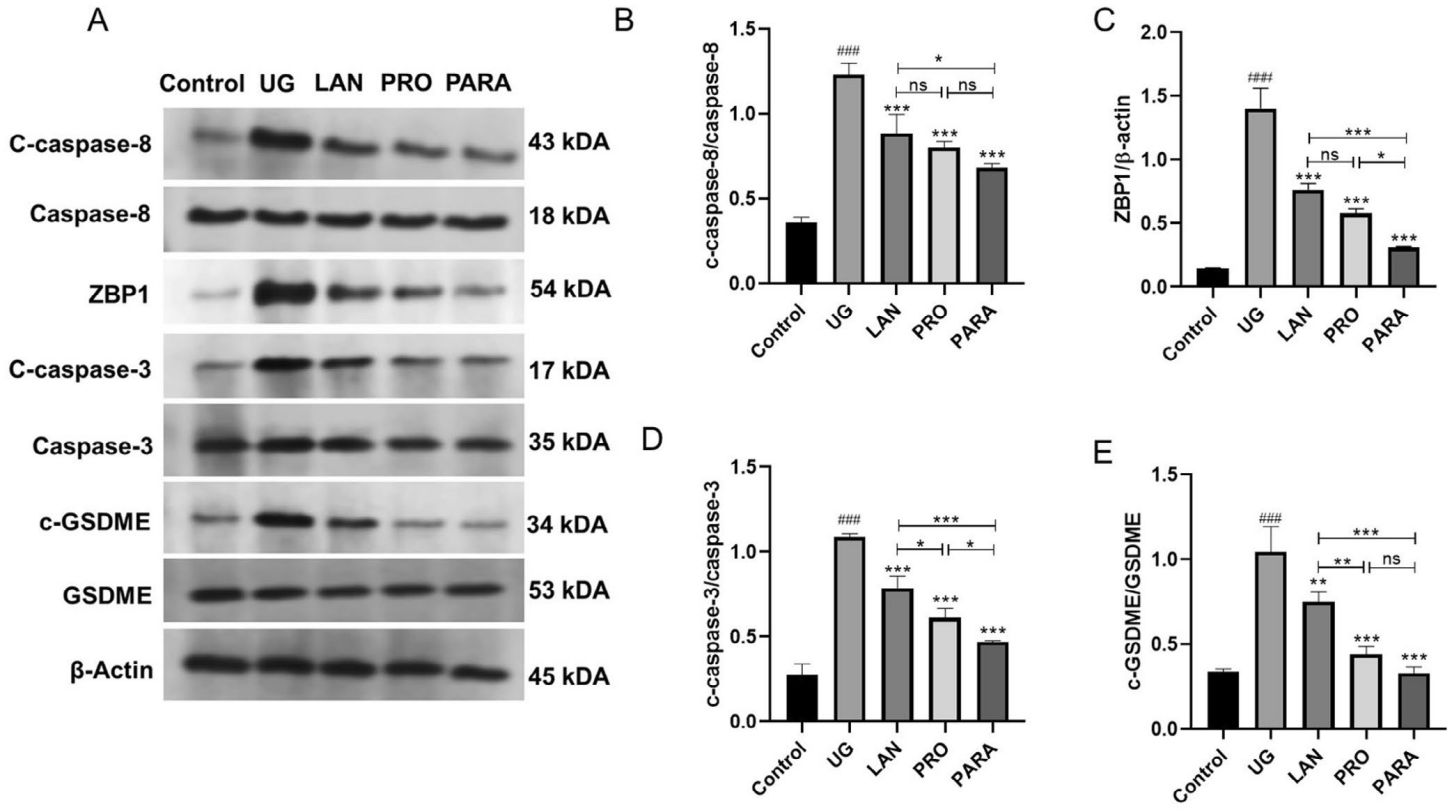
Effects of probiotics and paraprobiotics on apoptosis pathway proteins in stomach tissue. (A) Representative Western blot bands for Caspase‐8, cleaved Caspase‐8 (C‐caspase‐8), ZBP1, Caspase‐3, cleaved Caspase‐3 (C‐caspase‐3), Gasdermin E (GSDME), cleaved GSDME (c‐GSDME), and β‐Actin as a loading control. (B–E) Densitometric analysis results for the ratio of C‐caspase‐8/Caspase‐8, the normalized density of ZBP1 to β‐Actin, the ratio of C‐caspase‐3/Caspase‐3, and the ratio of c‐GSDME/GSDME, respectively. Data are presented as mean ± standard error of the mean (SEM) (*n* = 3 biological replicates per group). Statistical analysis was performed using one‐way ANOVA followed by Tukey's post hoc test. ^###^ *p* < 0.001, vs. Control group. *p < 0.05, **p < 0.01, ***p < 0.001, vs. Ulcer group (UG) or as indicated between groups. ns, not significant.

All treatment groups (lansoprazole, probiotic, paraprobiotics) significantly inhibited this indomethacin‐induced apoptotic activation compared to the UG. All three treatments markedly reduced the expression of ZBP1 and the levels of the active forms of caspase‐8, caspase‐3, and GSDME (*p* < 0.001). When compared among themselves, the probiotic and paraprobiotics administrations were found to be superior to the reference drug lansoprazole in suppressing apoptosis. Notably, the paraprobiotics treatment exhibited the most potent anti‐apoptotic effect, suppressing both ZBP1 expression and caspase‐3 activation to a statistically significantly greater extent than even the live PRO group (*p < 0.05). These findings suggest that the gastroprotective effects of probiotics, and particularly paraprobiotics, are closely associated with their ability to effectively inhibit the ZBP1‐mediated apoptotic cell death cascade.

### 3.8 Inhibition of the Necroptosis Pathway by Probiotics and Paraprobiotics

The phosphorylation status of the core regulatory proteins of necroptosis, RIPK3 and MLKL, was evaluated by Western blot analysis to elucidate the involvement of the necroptosis pathway, the final major component of PANoptosis, in indomethacin‐induced gastric damage and the effects of the treatments on this pathway (Figure 8). The Western blot results indicated that the necroptosis signaling pathway was potently activated in the UG. Compared to the control group, the ratio of the active, phosphorylated form of RIPK3 (p‐RIPK3) to total RIPK3 (Figure 8B) and the ratio of the active, phosphorylated form of the necroptosis executioner molecule MLKL (p‐MLKL) to total MLKL (Figure 8C) were statistically significantly increased in the UG (^###^ *p* < 0.001).

**FIGURE 8:**
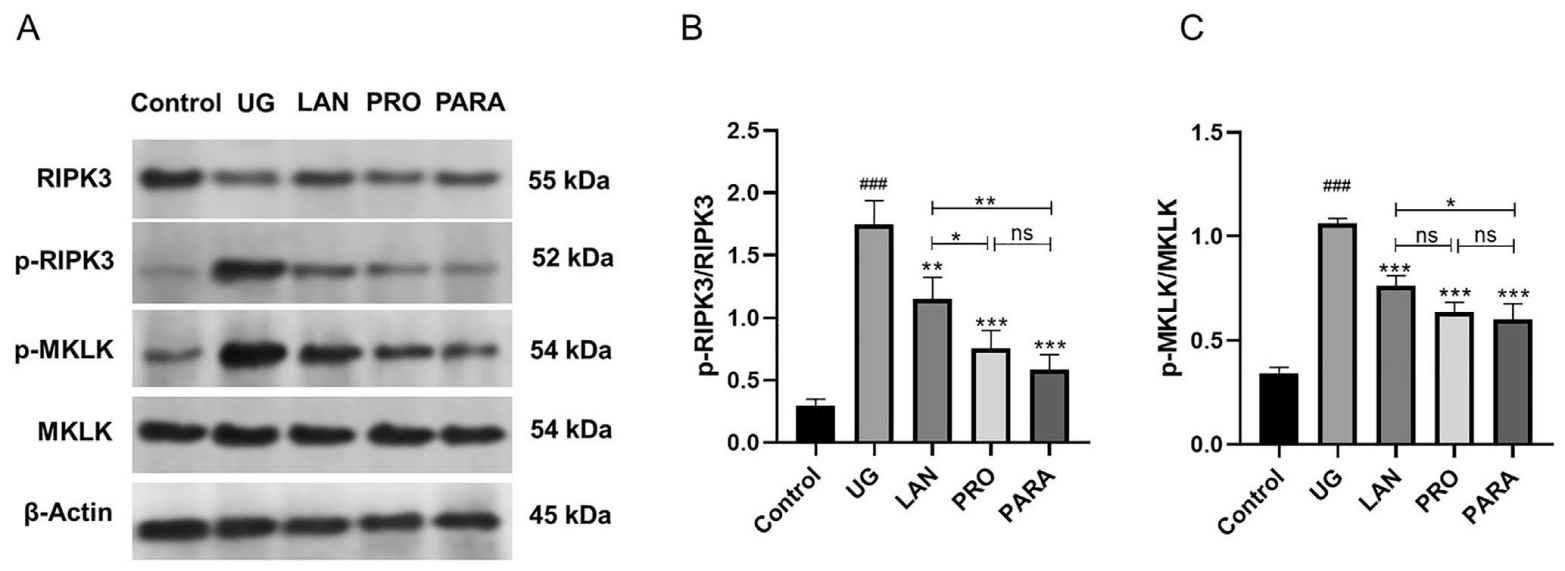
Effects of Probiotics and Paraprobiotics on Necroptosis Pathway Proteins in Stomach Tissue. (A) Representative Western blot bands for RIPK3, phospho‐RIPK3 (p‐RIPK3), phospho‐MLKL (p‐MLKL), MLKL, and β‐Actin as a loading control. (B) Densitometric analysis of the p‐RIPK3/RIPK3 ratio. (C) Densitometric analysis of the p‐MLKL/MLKL ratio. Data are presented as mean ± standard error of the mean (SEM) (*n* = 3 biological replicates per group). Statistical analysis was performed using one‐way ANOVA followed by Tukey's post hoc test. ^###^ *p* < 0.001, vs. Control group. *p < 0.05, **p < 0.01, ***p < 0.001, vs. Ulcer group (UG) or as indicated between groups. ns, not significant.

In contrast, all treatment groups (lansoprazole, probiotic, paraprobiotics) significantly suppressed this necroptotic activation compared to the UG. Both p‐RIPK3 and p‐MLKL levels were significantly reduced in all three treatment groups (*p* < 0.05, *p* < 0.01, or *p* < 0.001). A comparative analysis among the treatment groups revealed that the probiotic and paraprobiotics administrations were more effective than lansoprazole in inhibiting the necroptosis pathway. Specifically, p‐RIPK3 levels were statistically significantly lower in the PRO and PARA groups than in the LAN group (*p < 0.05 and **p < 0.01, respectively). Similarly, p‐MLKL levels were also found to be significantly lower in the PRO group than in the lansoprazole group (*p < 0.05). These molecular data provide strong support that the gastroprotective mechanisms of probiotics and paraprobiotics also involve their ability to effectively block the RIPK3‐MLKL‐mediated necroptotic cell death pathway, a critical arm of PANoptosis.

## 4 Discussion

Gastric ulcer is a complex pathology resulting from an imbalance between aggressive and protective factors, fundamentally driven by intense inflammation, oxidative stress, and widespread cell death. Although current pharmacological approaches focus on suppressing acid secretion, they are inadequate in targeting the underlying cellular and molecular damage mechanisms. This study comprehensively demonstrated the gastroprotective effects of the well‐documented probiotic *B. lactis* BB‐12 and its heat‐inactivated paraprobiotics derivative in an acute indomethacin‐induced gastric ulcer model. The most fundamental and innovative finding of our study is that this protective efficacy is closely associated with the potent inhibition of PANoptosis, a recently defined inflammatory cell death pathway that integrates pyroptosis, apoptosis, and necroptosis.

Flow cytometry analysis revealed that heat treatment at 85°C for 15 min completely inactivated the bacterial population by disrupting the membrane integrity of over 99% of the cells. This result agrees with previous reports showing that heat‐inactivated *Bifidobacterium animalis* subsp. *lactis* BB‐12 retains its structural integrity and functional potential as a paraprobiotics (Almada et al. 2021; Yolmeh et al. 2024). Similarly, our earlier work demonstrated that heat treatment fully inactivated *Lactobacillus acidophilus* LA‐5, whereas ultrasound produced viable but non‐culturable (VBNC) cells. Consequently, heat‐derived postbiotics exhibited stronger antimicrobial and probiotic‐stimulatory properties (Müldür et al. 2025). The importance of this methodological validation is significant because it establishes that the biological effects we observed in the PARA group originate not from live, metabolically active cells, but from non‐viable bacterial cells with largely preserved structural integrity. This underpins the advantages that paraprobiotics offer over live probiotics, such as a superior safety profile (especially for immunocompromised individuals), ease of standardization, and a long shelf life, making them a more viable candidate for clinical application (Shripada et al. 2020; Panitsidis et al. 2023).

Our findings at the macroscopic and histopathological levels clearly demonstrated that both probiotic and paraprobiotics *B. lactis* BB‐12 provide potent protection against indomethacin‐induced ulcerative damage. The significant reductions in ulcer area and damage score underscore the capacity of these agents to preserve mucosal integrity. These results support the extensive body of literature showing the gastroprotective effects of various probiotic strains in NSAID‐ or ethanol‐induced ulcer models. Gelen et al. (2024) showed that a probiotic mixture containing *Lactobacillus rhamnosus*, *L. fermentum* , and *L. brevis* provided significant gastroprotective effects in indomethacin‐induced gastric ulcers. In another study, Gelen et al. (2021) demonstrated that probiotic administration prevented indomethacin‐induced gastric mucosal damage, providing a marked gastroprotective effect. Similarly, Aliouche et al. (2024) showed that *Lactiplantibacillus plantarum* E1K2R2 and its exopolysaccharides exerted a strong gastroprotective effect in an ibuprofen‐induced acute gastric ulcer model. The results of our study showed that paraprobiotics derived from *B. lactis* BB‐12 exhibited a protective effect at least as potent as that of its live form, whereas at the macroscopic level it achieved a significantly lower damage score than the live probiotic. This indicates that non‐viable probiotics have remarkable potential to prevent gastric ulcers, comparable to their live counterparts. Unlike the long‐term colonization required for traditional probiotic efficacy, the rapid protection observed here suggests that paraprobiotics may primarily function through immediate mucosal interaction and receptor modulation, offering a distinct acute cytoprotective capability. It should also be emphasized that the effect of the treatments was not confined to the 5‐min pre‐dosing interval; the animals were sacrificed only 6 h after indomethacin administration, and the gastric mucosa is known to undergo pronounced temporal remodeling of inflammatory and cell‐death protein expression within this early window. In the indomethacin model, TNF‐α upregulation is the earliest, primary event that sequentially drives NF‐κB activation, MAP‐kinase signaling, and neutrophil‐derived myeloperoxidase during the early inflammatory response (Yadav et al. 2012); agents delivered at the onset of insult can therefore substantially reshape the expression of these downstream inflammatory and apoptotic proteins within the early hours of injury, as directly demonstrated for a gastroprotective agent delivered at the onset of injury and assessed at an early end‐point in this same 25 mg/kg indomethacin model (Arabacı Tamer et al. 2026). Similarly, literature has shown positive outcomes in digestive system diseases from studies involving postbiotics, which contain metabolites of probiotics but not live cells. Abbasi et al. (2022) emphasized that postbiotics represent a promising alternative to live probiotics for the prevention and treatment of gastrointestinal disorders. Gao et al. (2019) demonstrated that HM0539, a novel postbiotic protein derived from *Lactobacillus rhamnosus* GG, strengthens intestinal barrier integrity, thus holding protective potential against diseases associated with barrier dysfunction.

At the core of the pathogenesis of NSAID‐induced gastric damage lies oxidative stress, characterized by the excessive production of ROS and the failure of endogenous antioxidant defense mechanisms (Adem and Selim 2025). In our study, we found that indomethacin administration caused severe oxidative damage in the tissue by depleting the levels of vital antioxidants such as SOD, CAT, and GSH, and increasing the levels of MDA, an indicator of lipid peroxidation. Both probiotic and paraprobiotic treatments effectively reversed this oxidative imbalance. The upregulation of antioxidant enzyme activities and GSH levels suggests that these agents not only act as ROS scavengers but also bolster the cell's own antioxidant defense system. The successful suppression of MPO activity, a marker of neutrophil infiltration and a major source of ROS production, by both treatments highlights the multifaceted nature of this protective effect. The treatments not only mitigated the resulting oxidative damage but also inhibited the inflammatory cell migration that is a primary cause of this damage. Isik et al. (2022) reported that probiotics protect mucosal integrity in the gastrointestinal system by suppressing oxidative stress and inflammation, thus holding protective potential against NSAID‐induced mucosal injuries like ulcers. Similarly, Gelen et al. (2024) reported that *Lactobacillus rhamnosus* probiotic provides a gastroprotective effect in gastric ulcers by reducing oxidative stress and suppressing the inflammatory response. These findings in the literature support the antioxidant effect demonstrated by both probiotic *B. lactis* BB‐12 and its paraprobiotic form. In line with our observations, a series of recent in vivo studies has reinforced that restoring the SOD/CAT/GSH antioxidant axis while curbing lipid peroxidation and pro‐inflammatory cytokine release is central to gastric ulcer protection, whether the protective agent is a plant‐derived compound or a microbial preparation (Ahmed et al. 2026; Ahmed, Alqaisi, Ibrahim, et al. 2025; Ahmed, Alqaisi, Jabbar, et al. 2025; Al‐Qaisi et al. 2025a, 2025b).

Gastric ulcer is not merely a local event but also triggers a systemic inflammatory response (Saad and Qutob 2023). Our findings showed a dramatic increase in the serum levels of pro‐inflammatory cytokines TNF‐α and IL‐6 and a sharp decrease in the anti‐inflammatory cytokine IL‐10 in the UG, suggesting a predominance of a Th1‐type immune response in the pathogenesis. Both probiotic and paraprobiotic administrations reprogrammed this cytokine profile toward an anti‐inflammatory direction, exerting a potent immunomodulatory effect. The efficacy of paraprobiotics in reducing TNF‐α and IL‐6 levels underscores how powerful the systemic anti‐inflammatory potential of even non‐viable bacterial components can be. The suppression of IL‐10 levels by both treatments indicates that these therapies not only suppress destructive inflammation but also actively promote the anti‐inflammatory and immunoregulatory processes critical for tissue repair and regeneration. Saad and Qutob (2023) reported that an increase in serum IL‐6 and TNF‐α levels in experimental ulcer models leads to changes in hematological and hemostatic parameters, indicating that gastric ulcer triggers not only a local but also a systemic inflammatory response. Mehta et al. (2025) reported that *Streptococcus lutetiensis* isolated from traditional fermented food, as well as its paraprobiotic and postbiotic forms, exhibit immunomodulatory effects by regulating cytokine profiles, thus suggesting they could be safe biotherapeutics as an alternative to live probiotics. Likewise, Beiranvand (2022) reported that probiotics reduce ulcer development by strengthening the gastric mucosal barrier and suppressing inflammatory cytokines.

The most innovative and central finding of our study is its explanation of the gastroprotective effects of the treatments through the intersection of programmed cell death pathways, PANoptosis (Sun et al. 2024). Our western blot analyses revealed that indomethacin does not cause random cell death in the stomach tissue but rather specifically and coordinately activates the three major arms of PANoptosis. First, the marked increase in the activation of the NLRP3 inflammasome, Caspase‐1, and its substrates GSDMD and IL‐1β in the UG indicated that pyroptosis, an inflammatory form of cell death, plays an active role in the pathogenesis. Pyroptosis not only causes lytic cell death but also exacerbates inflammation through the release of IL‐1β (Qu et al. 2020). The effective inhibition of this pathway by our treatments, particularly the paraprobiotics, starting from the NLRP3 level, is a critical mechanism for breaking the inflammatory cycle. Selim et al. (2023) showed that NLRP3‐mediated pyroptosis exacerbates ulcer damage in an ethanol‐induced gastric ulcer model, while fucoidan treatment provides a gastroprotective effect by suppressing this process. Furthermore, Ji et al. (2025) discussed the critical role of pyroptosis as an inflammatory cell death mechanism in mucosal damage‐related diseases, including gastric ulcer.

Second, the increase in the activation of the PANoptosis sensor ZBP1 and the subsequent initiator Caspase‐8 and executioner Caspase‐3 demonstrated that the classic apoptosis cascade was also triggered. More interestingly, Gasdermin E (GSDME), a substrate of Caspase‐3, was also cleaved and activated. Based on current mechanistic models, the cleavage of GSDME is thought to convert apoptosis, normally a “silent” form of cell death, into a secondary (lytic) necrosis through pore formation, resembling pyroptosis; in the present study this axis was inferred from the observed changes in GSDME cleavage rather than being demonstrated directly at the level of pore formation. This illustrates how the apoptosis and pyroptosis pathways are intertwined in the pathogenesis of gastric ulcers. The potent suppression of both ZBP1 expression and the Caspase‐3/GSDME axis by our treatments, especially the paraprobiotics, reveals that they prevent both classic apoptosis and its transformation into an inflammatory form. Khuu (2024) highlighted that *H. pylori* infection can activate the ZBP1 sensor by suppressing classic cell death pathways in neutrophils, which may play a critical role in the pathogenesis of gastritis and peptic ulcer. Zhao and Lin (2025) emphasized that PANoptosis can be activated via the ZBP1 sensor in the intestinal epithelium, where Caspase‐3 cleaves GSDME to amplify the pyroptotic response, thereby contributing to the severity of mucosal inflammation. The regulation of these pathological pathways by probiotics and paraprobiotics resulted in a significant gastroprotective effect.

Third, the increase in the phosphorylation of RIPK3 and its substrate MLKL in the indomethacin‐induced ulcer model showed that necroptosis, a form of programmed necrosis, also plays a significant role in pathogenesis. Necroptosis is a death pathway that can be activated as an escape route when apoptosis is blocked, triggering an intense inflammatory response through the uncontrolled release of cellular contents (Ye et al. 2023). The effective inhibition of the activation of these kinases by both of our treatments (probiotic and paraprobiotic) indicates that they prevent the lytic rupture of cells, thereby stopping the further spread of inflammation. Liu et al. showed that high concentrations of ethanol trigger RIP1‐mediated necroptosis in gastric epithelial cells, independent of apoptosis, and that this could be a potential target for the treatment of acute alcohol‐induced gastric damage (Liu et al. 2021).

The simultaneous activation of these three major cell death pathways (pyroptosis, apoptosis, and necroptosis) suggests that PANoptosis could be a therapeutic target in indomethacin‐induced gastric ulcer pathology. This is pioneering evidence suggesting that gastric ulcer is driven not by a single cell death mechanism but by this integrated and complex death network. In this context, the most striking and holistic conclusion of our study is that probiotics, and especially paraprobiotics, can simultaneously and effectively inhibit all three arms of this death network. This provides a potential molecular basis for their potent gastroprotective effect. A treatment targeting only apoptosis or only necroptosis might be insufficient because the other pathways would remain active, whereas paraprobiotics, by simultaneously modulating different starting points like NLRP3, ZBP1, and RIPK3, act as broad‐spectrum cell death regulators that stop intracellular stress and death signals at their source. Similarly, Huo et al. emphasized that a prognostic signature based on pyroptosis, apoptosis, and necroptosis (PAN) subtypes in gastric cancer is closely associated with patient survival and immune response, and therefore, programmed cell death pathways should be considered in clinical management (Huo et al. 2022). Khuu et al. suggested that *H. pylori* infection is the main cause of gastritis and peptic ulcer, and that it can trigger inflammatory cell death pathways like pyroptosis, apoptosis, and necroptosis during this process; therefore, PANoptosis may also play a role in ulcer pathogenesis (Khuu 2024).

Despite the significant findings of this study revealing the effects of paraprobiotics on the PANoptosis pathway, there are some limitations that should be considered when interpreting the results. First, our study focused on a single acute gastric ulcer model (indomethacin‐induced) and a single probiotic strain ( *B. lactis* BB‐12). Therefore, the generalizability of the findings to ulcer models with different etiologies, such as *H. pylori* infection or stress, and whether the observed effects are strain‐specific, remain uncertain. Since it is known that different probiotic strains can interact with the host in different ways, these effects need to be investigated with a broader range of probiotics and paraprobiotics. Second, PANoptosis is a highly complex network, and this study only examined the expression of key regulatory proteins. To obtain a more holistic picture of this pathway, further investigation of other sensors and adaptor proteins that form the PANoptosome complex, such as ASC and FADD, at both the protein and transcriptional (gene) levels, will provide a deeper understanding of the mechanism. Furthermore, while the current in vivo study utilized a 6‐h interval to capture the peak phase of macroscopic mucosal injury and the integrated physiological response, including mucus barrier function and systemic immunity, future mechanistic studies incorporating in vitro models would be valuable for refining the pharmacokinetic/pharmacodynamic (PK/PD) window and dissecting the precise temporal kinetics of these agents. Finally, our study does not include a direct statistical correlation analysis between the inflammatory markers in the stomach tissue and the expression of proteins in the PANoptosis pathway. Performing such an analysis would further strengthen the causal relationship between the observed anti‐inflammatory effects and the inhibition of the cell death pathway. These limitations, while highlighting important directions for future research, do not diminish the significance of the topic.

## 5 Conclusion

This study demonstrates that both the probiotic *B. lactis* BB‐12 and its paraprobiotics derivative provide potent gastroprotection in an indomethacin‐induced ulcer model, with the paraprobiotics showing comparable or superior efficacy. Crucially, we reveal for the first time that this protective mechanism is closely associated with the broad inhibition of the PANoptosis cell death network, simultaneously blocking pyroptosis, apoptosis, and necroptosis. These findings validate paraprobiotics as a safer, more stable therapeutic alternative for inflammatory diseases like gastric ulcer and establish PANoptosis as a promising new target for future gastroprotective strategies. Therefore, future preclinical investigations are imperative to delineate the precise upstream molecular mechanisms driving PANoptosis inhibition, while concurrently establishing a comprehensive safety profile and an optimal dosing strategy in diverse models of gastric injury.

## Author Contributions

**Adem Yavaş:** conceptualization, data curation, methodology, supervision, investigation, visualization, formal analysis, writing – original draft. **Ezgi Özkiran:** methodology, writing – review and editing. **Ecem Akan:** methodology, formal analysis, writing – review and editing.

## Funding

AY acknowledges financial support from the Scientific and Technological Research Council of Türkiye (TUBITAK) under project number 125Z143. The article processing charge was supported by the Scientific Research Coordination Unit of Aydın Adnan Menderes University under project number ÇMYO‐26001.

## Disclosure

The authors declare that they have not used AI‐generated work in this manuscript.

## Conflicts of Interest

The authors declare no conflicts of interest.

## Data Availability

The data that support the findings of this study are available from the corresponding author upon reasonable request.
